# Health-related quality of life in primary hepatic cancer: a systematic review assessing the methodological properties of instruments and a meta-analysis comparing treatment strategies

**DOI:** 10.1007/s11136-021-02810-8

**Published:** 2021-07-20

**Authors:** Kerstin Wohlleber, Patrick Heger, Pascal Probst, Christoph Engel, Markus K. Diener, André L. Mihaljevic

**Affiliations:** 1 Department of General, Visceral and Transplant Surgery, University of Heidelberg, Im Neuenheimer Feld 110, 69120 Heidelberg, Germany; 2 The Study Centre of the German Surgical Society (SDGC), University of Heidelberg, Im Neuenheimer Feld 110, 69120 Heidelberg, Germany; 3 Institute for Medical Informatics, Statistics and Epidemiology (IMISE), University of Leipzig, Härtelstraße 16-18, 04107 Leipzig, Germany

**Keywords:** Quality of life, Health-related quality of life, Hepatocellular carcinoma, Cholangiocellular carcinoma

## Abstract

**Purpose:**

Patient-reported outcomes including health-related quality of life (HRQoL) are important oncological outcome measures. The validation of HRQoL instruments for patients with hepatocellular and cholangiocellular carcinoma is lacking. Furthermore, studies comparing different treatment options in respect to HRQoL are sparse. The objective of the systematic review and meta-analysis was, therefore, to identify all available HRQoL tools regarding primary liver cancer, to assess the methodological quality of these HRQoL instruments and to compare surgical, interventional and medical treatments with regard to HRQoL.

**Methods:**

A systematic literature search was conducted in MEDLINE, the Cochrane library, PsycINFO, CINAHL and EMBASE. The methodological quality of all identified HRQoL instruments was performed according to the COnsensus-based Standards for the selection of health status Measurements INstruments (COSMIN) standard. Consequently, the quality of reporting of HRQoL data was assessed. Finally, wherever possible HRQoL data were extracted and quantitative analyses were performed.

**Results:**

A total of 124 studies using 29 different HRQoL instruments were identified. After the methodological assessment, only 10 instruments fulfilled the psychometric criteria and could be included in subsequent analyses. However, quality of reporting of HRQoL data was insufficient, precluding meta-analyses for 9 instruments.

**Conclusion:**

Using a standardized methodological assessment, specific HRQoL instruments are recommended for use in patients with hepatocellular and cholangiocellular carcinoma. HRQoL data of patients undergoing treatment of primary liver cancers are sparse and reporting falls short of published standards. Meaningful comparison of established treatment options with regard to HRQoL was impossible indicating the need for future research.

**Supplementary Information:**

The online version contains supplementary material available at 10.1007/s11136-021-02810-8.

## Introduction

Besides survival and treatment-associated adverse events, patient-reported outcomes (PROs) are arguably the most relevant outcome parameters in oncology. A PRO is defined as ‘any outcome evaluated directly by the patient himself or herself and is based on patient’s perception of a disease and its treatment(s)’ [1]. PROs have many potential advantages as they may elucidate the relationship between clinical endpoints and the patient´s well-being [1], allowing for a more comprehensive evaluation of patients’ health [2].

Health-related quality of life (HRQoL) is a multidimensional PRO measure that is of special interest in oncology as it provides a ‘personal assessment of the burden and impact of a malignant disease and its treatment,’ [1] thus, adding valuable information for a true risk–benefit assessment. This is of special interest when prognosis is limited as in primary malignancies of the liver. HRQoL tools can be distinguished into generic, cancer-specific, cancer-type-specific and utility-(preference-)based instruments [3]. While definitions, implementation, evaluation and analyses of survival and toxicity/complication endpoints have been well standardized over the last decades, PROs are still under-evaluated and reported in most clinical settings. Multiple studies have aimed to define suitable HRQoL tools for different clinical settings, e.g. [4, 5], including cancer patients [6–8].

Hepatocellular carcinoma (HCC) and intrahepatic cholangiocarcinoma (CCA) account for more than 95% of all primary malignant liver tumours. Hepatitis B and C infections are the most prominent risk factor for HCC [9]. More than 840.000 patients were newly diagnosed with HCC or CCA in 2018, and numbers are estimated to rise > 1.3 million annually until 2040 [10]. Although age-standardized incidence rates are moderate in the Western World, they are high in most parts of Asia and parts of West Africa [10], making HCC one of the most frequent tumours in these parts of the world. Prognosis is dismal with 5-year overall survival being around 15% in the USA and 5% in low-income countries [9]. Besides surgical resection, medical treatment (e.g. chemotherapy, kinase inhibitors) and interventional treatments like radiofrequency ablation (RFA) and transarterial chemoembolization (TACE) constitute the three mainstays of treatment for both HCC and CCA.

Therefore, the objectives of this systematic review and meta-analysis were threefold: (1) to perform a systematic review to identify all published HRQoL tools for primary liver cancer (HCC/CCA); (2) to assess the methodological quality and clinical relevance of these HRQoL measures; and (3) to synthesize quantitative data via means of a meta-analysis to compare surgery vs. interventional treatments vs. systemic therapies with regard to HRQoL.

## Material and methods

This systematic review and meta-analysis is reported in line with current PRISMA guidelines [11]. The study was registered in the PROSPERO database on 18th July 2017 (registration number CRD42017068103).

### Eligibility criteria

Studies investigating HRQoL in HCC or CCA patients were included independent of language or year of publication. All types of studies were included in our search with the exception of case reports, i.e. randomized controlled trials (RCT), cohort-type studies (CTS), case–control studies (CCS) and cross-sectional studies. Furthermore, studies in animals (non-human studies) were excluded. The patient (P) and outcome (O) terms of the PICOT (patient–intervention–comparison–outcome–time) scheme were used to build a search strategy. The search used the ‘outcome’ term to identify PROMs describing quality of life or HRQoL and the ‘patient’ term to find studies including patients with HCC or CCA. Supplement 1 shows the search strategy for MEDLINE performed via OvidSP. If studies included mixed patient populations (e.g. including HCC patients together with metastatic cancer patients and other tumours), only those trials were included in which HRQoL data could clearly be extracted for HCC and CCA patients.

### Information sources

The following databases were searched [12]: (a) MEDLINE via OvidSP last searched on 18th July 2019; (b) Ovid MEDLINE In-Process & Other Non-Indexed Citations via OvidSP last searched on 18th July 2019; (c) the Cochrane library (including Cochrane reviews, other reviews, trials, technology assessments and economic evaluations) via the Cochrane homepage (Wiley online library) last searched on 18th July 2019; (d) PsycINFO via EBSCO host last searched on 18th July 2019; (e) CINAHL via EBSCO host last searched on 18th July 2019 and (f) Excerpta Medica Database (EMBASE) via EMBASE homepage last searched on 18th July 2019. The references of the included articles were hand searched to identify additional relevant studies. Where necessary, authors were directly contacted to retrieve missing information.

### Search

Sensitive search strategies were developed for all databases using wildcards and adjacency terms where appropriate. Supplement 1 shows the search strategy for MEDLINE performed via OvidSP. The search strategies for the other databases were adapted to the specific vocabulary of each database.

### Study selection

**Fig. 1:**
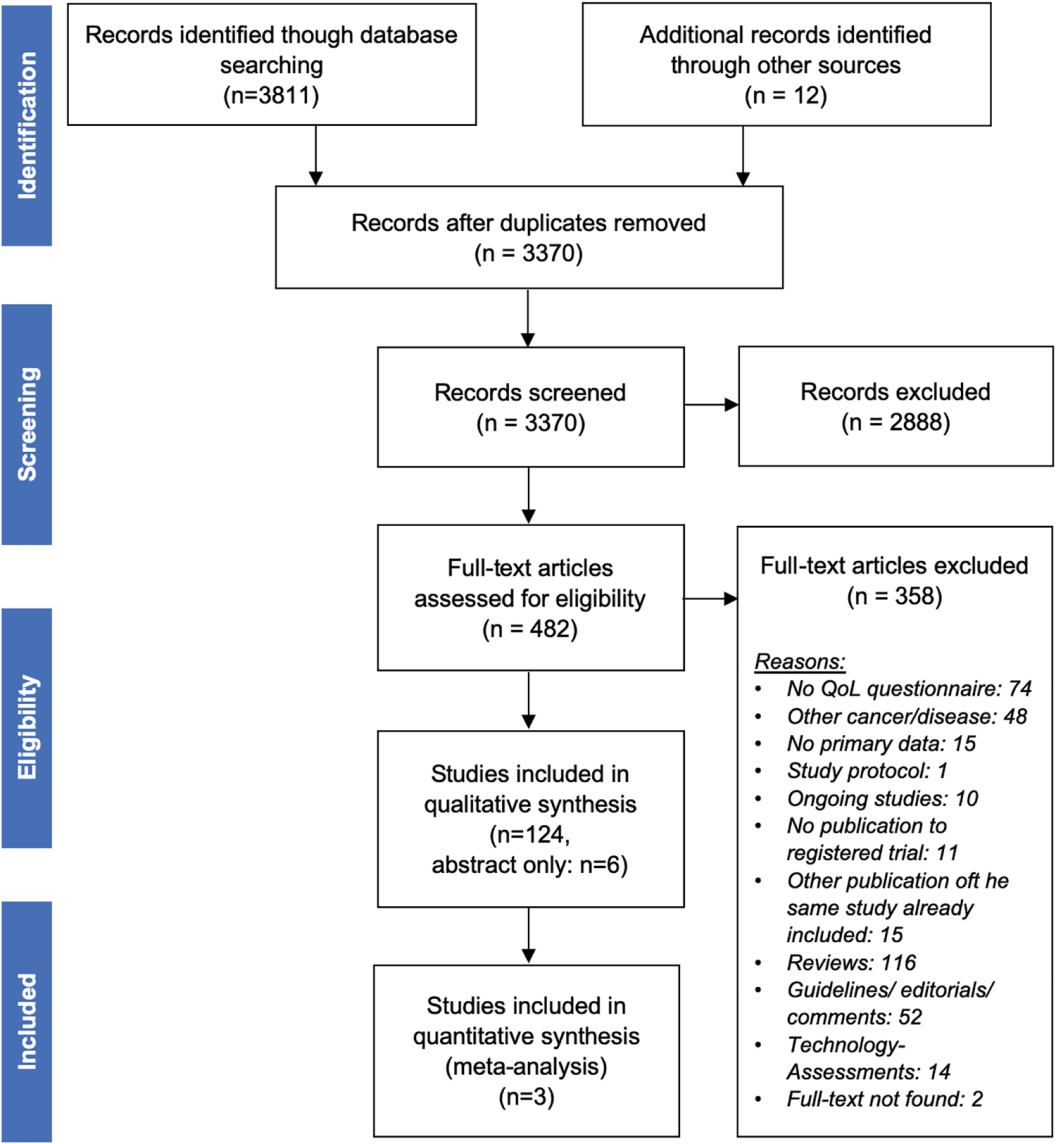
Flow chart of included studies

Search results were imported into EndNote software (EndNote X7.7, Thomson Reuters) [13], and duplicates were removed by using the automated duplicate removal function of EndNote. Consequently, titles and abstracts of studies were screened by two authors (KW, ALM) for fulfilment of inclusion and exclusion criteria. Remaining duplicates were removed manually. For the remaining studies, full text articles were obtained, which were then screened for eligibility by two authors independently (KW, ALM). Reasons for exclusion of full text articles were recorded (Fig. 1). All remaining articles were included in the qualitative syntheses (objectives 1 and 2). For objective 3 (quantitative assessment), all articles using adequate HRQoL measures (i.e. fulfilling objective 2) were included in the assessment of quality of reporting of HRQoL data and risk of bias assessment of individual studies. HRQoL data were extracted wherever possible and grouped according to the three clinical settings: (a) surgery; (b) interventional therapy and (c) medical treatment.

HRQoL assessments were then grouped into 3-month periods. In a next step, quantitative data analysis was performed for those HRQoL measures for which ≥ 2 quantitative data time points were available. For quantitative data analysis, results of individual studies were entered in RevMan 5 software 5.3. (Review Manager, Version 5.3 Copenhagen: The Nordic Cochrane Center, The Cochrane Collaboration, 2014).

### Data collection process

Data were extracted by two authors independently (KW, ALM) and collected on pre-specified piloted forms. In case, required data were not reported in the study, and authors were contacted to obtain remaining data. Differences in data extraction were resolved by consensus with a third author (MKD).

### Data items

The following data items were collected: title, author, year of publication, country where study was performed, journal, language, cancer type, intervention, control, co-interventions, primary endpoint, secondary endpoints, HRQoL tool used, type of study, number of centres, start and end dates of study and intervention, number of patients (total), number of patients allocated to intervention(s), number of patients allocated to control, number of patients evaluated for HRQoL (at each point in time), number of withdrawals, exclusions, conversions, duration of follow-up, HRQoL data at baseline and during follow-up, analysis strategy, subgroups measured and subgroups reported. Furthermore, the following baseline characteristics of patients (for both intervention and control group) were recorded: age, gender, severity of illness, co-morbidities and other relevant baseline characteristics.

### Evaluation of methodological quality of the HRQoL measures

The methodological quality of HRQoL measures was assessed based on specific psychometric criteria. Owing to the lack of uniform consensus on how to appraise PRO measures, criteria were applied based on published recommendations [3, 14] in accordance with U.S. Food and Drug Administration guidance [15] and the Oxford University PROMs Group guidelines and the COnsensus-based Standards for the selection of health status Measurements INstruments (COSMIN) [16]. The criteria and benchmarks laid out in Table 1 were used for evaluation and have been used in previous publications [4, 5]. A rating scale described in previous publications was applied to allocate a mark for each domain [4, 5]: 0 no evidence reported;—evidence not in favour; + evidence in favour; ± conflicting evidence. Lack of basic psychometric evaluation was defined by a priori consensus as evaluation of less than 2 positive ( +) aspects (other than feasibility and interpretability) in HCC/CCA patients. Evaluation was limited to primary hepatic cancers (HCC/CCA), i.e. the psychometric properties of some instruments might have been evaluated in other types of cancer, but not in HCC/CCA patients. In case of lack of psychometric data for a given instrument, searches were conducted in Medline to identify additional studies that have evaluated the psychometric properties of the HRQoL instrument in closely related patient cohorts (e.g. patients with chronic liver disease).

**Table 1:** Psychometric criteria used to assess the quality of the patient-reported outcome measures

Domain	Criteria
Test–retest reliability	Test–retest: the intraclass correlation/weighted κ score should be ≥ 0.70 for group comparisons and ≥ 0.90 if scores are going to be used for decisions about an individual based on their score. The mean difference (paired *t* test or Wilcoxon signed-rank test) between time points 1 and 2, and the 95% CI should also be reported
Internal consistency	A Cronbach’s α score of ≥ 0.70 is considered good, and it should not exceed ≥ 0.92 for group comparisons as this is taken to indicate that items in the scale could be redundant. Item correlations should be ≥ 0.20
Content validity	This is assessed qualitatively during the development of an instrument. To achieve good content validity, there must be evidence that the instrument has been developed by consulting patients and experts as well as undertaking a literature review. Patients should be involved in the development stage and item generation. The opinion of patient representatives should be sought on the constructed scale
Construct validity	A correlation coefficient of ≥ 0.60 is taken as strong evidence of construct validity. Authors should make specific directional hypotheses and estimate the strength of correlation before testing
Criterion validity	A good argument should be made as to why an instrument is standard and correlation with the standard should be ≥ 0.70
Responsiveness	There are a number of methods to measure responsiveness, including t tests, effect size, standardized response means or Guyatt’s responsiveness index. There should be statistically significant changes in score of an expected magnitude
Appropriateness	Assessment whether the content of the instrument is appropriate to the questions which the clinical trial is intended to address
Interpretability	Subjective assessment whether the scores of the instrument are interpretable for patients or physicians
Acceptability	Acceptability is measured by the completeness of the data supplied; ≥ 80% of the data should be complete
Feasibility	Qualitative assessment whether the instrument is easy to administer and process
Floor-Ceiling effect	A floor or ceiling effect is considered if 15% of respondents are achieving the lowest or the highest score on the Instrument

Adapted from [4, 5]

### Evaluation of the quality of reporting of HRQoL data

For assessment of reporting, the studies were analysed using the following questions: (a) Is HRQoL data analysis described in methods section? (b) Has an a priori statistical analysis plan for HRQoL outcomes been implemented, addressing common problems like missing data, multiple testing? (c) Is HRQoL raw data presented? (d) Is individual patient data reported? (e) Which summary scores are used for HRQoL data? (f) Which time points of HRQoL assessment are described in the methods section? g.) For which time points is HRQoL data reported in the results section?

### Assessment of risk of bias in individual studies

For RCTs risk of bias was judged using The Cochrane Collaboration tool of for assessing quality and risk of bias [17]. Risk of bias for non-randomized, interventional trials was assessed with the ROBINS-I tool (Risk Of Bias In Non-randomized Studies—of Interventions, formerly known as ACROBAT-NRSI) as recommended by the Cochrane collaboration [11]. Non-randomized, non-interventional studies were assessed using the Newcastle–Ottawa risk of bias tool [18], and cross-sectional studies were assessed using the AHRQ checklist. RCTs were judged to be at an overall high risk of bias if there was a serious risk of bias in any of the following domains: random sequence generation, allocation concealment, missing data. For non-randomized trials, the following overall risk of bias judgement for individual studies was used in line with Cochrane recommendations [11]: (a) low risk of bias: the study is judged to be at low risk of bias for all domains; (b) moderate risk of bias: the study is judged to be at low or moderate risk of bias for all domains; (c) serious risk of bias: the study is judged to be at serious risk of bias in at least one domain, but not at critical risk of bias in any domain; (d) Critical risk of bias: the study is judged to be at critical risk of bias in at least one domain.

#### Statistical analysis

Data were entered in RevMan 5 software 5.3. (Review Manager, Version 5.3 Copenhagen: The Nordic Cochrane Center, The Cochrane Collaboration, 2014) [19]. As level of significance, an alpha of 0.05 was determined. A random-effect model (inverse variance) was used as there has been clinical heterogeneity between the included trials. Heterogeneity was evaluated using I^2^ statistic. Results lower than 25% were considered as low, between 25% and 75% as possibly moderate, and results of I^2^ over 60% were considered as a considerable heterogeneity. HRQoL in HCC/CCA patients was compared by meta-analysis for the following types of interventions: (a) surgery; (b) interventional therapies (e.g. TACE, RFA) and (c) systemic therapies (e.g. chemotherapy). Only studies using the FACT-G/FACT-Hep could be used for meta-analysis (see results section). As these subscores are continuous variables, the mean difference in the FACT-G/FACT-Hep subscores was used as effect measure.

## Results

### Study selection

We identified 3811 studies by database search and 12 additional studies by hand search resulting in a total of 3823 records. 453 of those studies were duplicates (Fig. 1). After screening titles and abstracts, the other 2888 records were excluded according to inclusion and exclusion criteria. Subsequently, the other 358 articles were excluded after full text analyses for the following reasons: no HRQoL tool (*n* = 74), other type of cancer (no HCC/CCA) (*n* = 48), no primary data (*n* = 198), ongoing study without report (*n* = 21), double publication (*n* = 15) and no full text available (*n* = 2). The remaining 124 studies were included in the final qualitative syntheses (Fig. 1).

### Study characteristics

The characteristics of the 124 included studies are listed in Table 2 [20–140]. Most studies were cohort-type studies (*n* = 50; 40.3%), either with (*n* = 12; 24%) or without control group (*n* = 38; 76%). The remaining studies were RCTs (*n* = 41; 33.1%), non-randomized controlled trials (*n* = 18; 14.5%), cross-sectional studies (*n* = 7; 5.6%) or case–control studies (*n* = 8; 6.5%) (Supplement 2). A total of 21,496 patients were included in all studies. Frequently studies investigated HCC patients only (Supplement 2). Most studies were single-centre studies (*n* = 83; 66.9%; supplement 2). The country of origin is depicted in Supplement 2.

**Table 2:** Baseline characteristics of the included studies

	Author	Type of cancer	Study type	Country	Number of centers	Type of intervention	Intervention	Number of patients	Study description	QoL also primary vs. Secondary endpoint	PROM	Comment
1	Saleh et al. [20]	HCC	RCT	Egypt	2	Interventional	RFA	32	RFA vs. hepatic resection	Secondary	Questionnaire by Abdelbary	
						Surgical	Liver resection	28				
2	Abou-Alfa et al. [21]	HCC	RCT	19 countries	95	Medical	Cabozantinib	470	Cabozantinib vs. placebo in patients with previous sorafenib therapy	Secondary	EQ-5D	
						Placebo	Placebo	237				
3	Aliberti et al. [22]	CCA	CTS	Italy	1	Interventional	TACE with Doxorubicin loaded beads	11	TACE with slow-Release Doxorubicin-Eluting Beads vs. palliative chemotherapy	Unclear	ESAS	
						Medical	Palliative CTx	9				
4	Barbare et al. [23]	HCC	RCT	France	78	Medical	Tamoxifen	210	Tamoxifen vs. best supportive care	Secondary	Spitzer QoL Index	
						Placebo	Best supportive care	210				
5	Barbare et al. [24]	HCC	RCT	France	79	Medical	Octreotide	135	Octreotide vs. placebo	Secondary	EORTC QLQ-C30	
						Placebo	Placebo	137				
6	Becker et al. [25]	HCC	RCT	Germany, Switzerland	7	Medical	Octreotide	61	Octreotide vs. placebo	Secondary	EORTC QLQ-C30	
						Placebo	Placebo	59				
7	Berr et al. [26]	CCA	CTS	Germany	1	Interventional	23	23	Photodynamic therapy + biliary stenting	Secondary	Spitzer QoL Index	
8	Bianchi et al. [27]	HCC	CCS	Italy	4	No intervention	None. Patients with HCC	101	Comparison of QoL in patients with HCC vs. liver cirrhosis	Primary	SF-36 + Nottingham Health Profile	
						No intervention	None. Patients with liver cirrhosis	202				
9	Blazeby et al. [28]	HCC	CTS	Great Britain, Hong Kong, Taiwan	6	Mixed	Mixed treatments	158	Development of the HCC18 supplement	Primary	EORTC QLQ-HCC18	
10	Boulin et al. [29]	HCC	NRCT	France	1	Interventional	15 mg Idarubicin	6	Phase II-Studies of TACE with DC-Beads loaded with idarubicin	Secondary	EORTC QLQ-C30	QoL data is reported in Anota et al. 2016
						Interventional	10 mg Idarubicin	6				
						Interventional	5 mg Idarubicin	9				
11	Brans et al. [30]	HCC	CTS	Belgium	1	Interventional	131I-lipiodol instillation	26	Instillation of 131I-Lipiodol in the proper hepatic artery during a hepatic angiography	Primary	EORTC QLQ-C30	
12	Bruix et al. [31]	HCC	RCT	21 countries	152	Medical	Regorafenib	374	Regorafinib vs. placebo in patients with disease progression under sorafenib	Secondary	FACT-G, FACT-Hep, EQ-5D	
						Placebo	Placebo	193				
13	Brunocilla et al. [32]	HCC	CTS	Italy	1	Medical	Sorafenib	36	Sorafenib treatment in patients with HCC	Secondary	FACT-Hep	
14	Cao et al. [33]	HCC	CTS	China	1	Interventional	TACE	155	TACE in patients with HCC	Primary	MDASI	
15	Cebon et al. [34]	HCC	CTS	Australia	13	Medical	Octreotide	63	Octreotide until tumour progression or toxicity	Unclear	FACT-Hep + Pt DATA Form	
16	Chang-Chien et al. [35]	HCC	CTS	Taiwan	3	Surgical	Surgery	284	QoL evaluation after surgical treatment for HCC	Primary	FACT-Hep + EORTC QLQ-C30 + SF-36	
17	Chay et al. [36]	HCC	RCT	Singapore	1	Medical	Coriolus versicolor	9	Coriolus versicolor vs. placebo	Secondary	EORTC QLQ-C30 + FACT-Hep	
						Placebo	Placebo	6				
18	Chen et al. [39]	HCC	CTS	China	1	Interventional	TACE	142	TACE (peripheric embolization)	Secondary	EORTC QLQ-C30	
19	Cheng et al. [38]	HCC	RCT	China, South Korea, Taiwan	23	Medical	Sorafenib	150	Sorafenib vs. placebo	Unclear	FACT-Hep + FHSI-8	
						Placebo	Placebo	76				
20	Cheng et al. [40]	HCC	RCT	23 countries	136	Medical	Sunitinib	530	Sunitinib vs. Sorafenib	Secondary	EQ-5D	
						Medical	Sorafenib	544				
21	Chie et al. [41]	HCC	CTS	Taiwan, UK, China Japan, Italy, France	6	Surgical	Surgical treatment	53	Cross-cultural validation study for EORTC QLQ-HCC18	Primary	EORTC QLQ-C30 + EORTC QLQ-HCC18	Chie et al. 2015 reports on the same data
						Interventional	Ablation	53				
						Interventional	Embolization	65				
						Medical	Systemic therapy	24				
						No intervention	Off-treatment	32				
22	Chie et al. [42]	HCC	CTS	Taiwan, UK, Italy, Japan, France	7	Mixed	Asian patients	181	Comparison of QoL in Asian vs. European HCC patients undergoing different types of treatments	Primary	EORTC QLQ-C30 + EORTC QLQ-HCC18	
						Mixed	European patients	46				
23	Chiu et al. [43]	HCC	CTS	Taiwan	3	Surgical	Hepatic resection	332	HCC patients that underwent hepatic resection	Primary	FACT-Hep + SF-36	
24	Chow et al. [44]	HCC	RCT	9 countries	10	Medical	Tamoxifen twice daily	120	Tamoxifen vs. tamoxifen + placebo vs. placebo	Secondary	EORTC QLQ-C30	
						Medical	Tanoxifen in the morning + placebo at night	74				
						Placebo	Placebo	130				
25	Chow et al. [45]	HCC	RCT	6 countries	7	Medical	Megestrolacetate	123	Megestrolacetate vs. placebo	Secondary	EORTC QLQ-C30	
						Placebo	Placebo	62				
26	Chow et al. [46]	HCC	CTS	4 countries	7	Medical	Sorafenib	29	Sorafenib 14 days post radio embolization	Secondary	EQ-5D	
27	Chung et al. [47]	HCC	CSS	Taiwan	3	Mixed	Mixed treatments	100	Symptom evaluation of HCC patients with different types of treatments	Primary	MDASI	
28	Cowawintaweewat et al. [48]	HCC	CTS	Thailand	1	Medical	Active Hexose Correlated Compound Treatment	34	AHCC vs. placebo	Primary	Questionnaire by Cowawintaweewat	
						Placebo	Placebo	10				
29	Darwish Murad et al. [49]	CCA	CTS	USA	1	Surgical	Neoadjuvant radio-chemotherapy + LT	79	Neoadjuvant radio-chemotherapy + LT for CCA vs. LT for other indication than CCA	Primary	EQ-5D + SF-36 + NIDDK-QA	
						Surgical	LT for other indication than CCA	110				
30	Dimitroulopoulos et al. [50]	HCC	NRCT	Greece	1	Medical	Positive ocreotide scan: Sandostatin	15	Sandostatin vs. no sandostatin	Secondary	EORTC QLQ-C30	
						Medical	Negative Octreoscan o refusing octreotide: no sandostatin	13				
31	Dimitroulopoulos et al. [51]	HCC	RCT	Greece	1	Medical	Octreoscan positive: octreotide s.c. and octreotide long-acting formulation	30	Octreotide vs. Placebo with positive Octreoscan compared to patients with negative Octreoscan	Secondary	EORTC QLQ-C30	
						Placebo	Octreoscan positive: placebo	30				
						Medical	Octreoscan negative: only follow-up	60				
32	Doffoël et al. [52]	HCC	RCT	France	15	Interventional	Tamoxifen + TACE	62	Tamoxifen + TACE vs. Tamoxifen	Secondary	Spitzer QoL Index	
						Medical	Tamoxifen	61				
33	Dollinger et al. [53]	HCC	RCT	Germany	12	Medical	Thymostimulin	67	Thymostimulin vs. placebo	Secondary	FACT-Hep	
						Placebo	Placebo	68				
34	Dumoulin et al. [54]	CCA	CTS	Germany	1	Interventional	Metal stent and photodynamic therapy	24	PDT vs. historic control	Unclear	EORTC QLQ-C30	
						No intervention	Historic control	20				
35	Eltawil et al. [55]	HCC + CCA	CTS	Canada	1	Interventional	TACE	48	TACE for primary liver cancer	Primary	WHOQoL-BREF	
36	Fan et al. [56]	HCC	CTS	Taiwan	2	Mixed	Surgery, TACE or systemic therapy	286	QoL of HCC patients treated with surgery, TACE or systemic therapy was compared to healthy norm values	Primary	EORTC QLQ-C30 + EORTC QLQ-HCC18	
37	Gill et al. [57]	HCC	CSS	13 countries	online- based	Mixed	Different treatments	256	All HCC patients were invited to complete the QoL survey	Primary	Questionnaire by Gill	
38	Gmur et al. [58]	HCC	CTS	Switzerland	1	Mixed	Different treatments	242	Evaluation of the predictive value of QoL on survival	Primary	FACT-Hep	
39	Guiu et al. [163]	HCC	NRCT	France	1	Interventional	Idarubicin 15 mg	4	Phase II study of TACE with DC-Beads with Idarubicin	Secondary	EORTC QLQ-C30	
						Interventional	Idarubicin 20 mg	4				
						Interventional	Idarubicin 25 mg	2				
40	Hakim et al. [59]	HCC	RCT	Zimbabwe	n.a	Medical	Adriamycin 20 mg weekly	112	Adriamycin vs. best supportive care	Unclear	FLIC	
						Medical	Adriamycin 80 mg monthly					
						No intervention	Best supportive care					
41	Hamdy et al. [60]	HCC	CTS	Egypt	1	Intervention 1	RFA	40	QoL compared in patients with HCC vs. chronic liver disease	Primary	SF-36	
						Intervention 2	TACE	40				
						Control	Patients with HCV but without HCC	40				
42	Hartrumpf et al. [61]	HCC	CTS	Germany	1	Interventional	TACE	148	TACE for patients with HCC	Primary	EORTC QLQ-C30 + EORTC QLQ-HCC18	
43	He et al. [62]	HCC	NRCT	China	1	Surgical	Liver transplantation	22	Liver transplantation vs. hepatic resection vs. RFA	Primary	SF-36	
						Surgical	Hepatic resection	68				
						Interventional	RFA	38				
44	Hebbar et al. [63]	HCC	RCT	France	17	Interventional	TACE + sunitinib	39	TACE + sunitinib vs. TACE + placebo	Secondary	unclear	
						Interventional	TACE + placebo	39				
45	Heits et al. [64]	HCC	CSS	Germany	1	Surgical	Liver transplantation	173	QoL in HCC patients after LT was compared to healthy norm values	Primary	EORTC QLQ-C30	
46	Hinrichs et al. [65]	HCC	CTS	Germany	1	Interventional	TACE	62	TACE for patients with HCC	Primary	EORTC QLQ-C30 + EORTC QLQ-HCC18	
47	Hoffmann et al. [66]	HCC	RCT	Germany	4	Medical	TACE + sorafenib	24	TACE + sorafenib vs. TACE + placebo until tumour progression or liver transplantation	Secondary	EORTC QLQ-C30 + EORTC QLQ-HCC18	QoL data is reported in Hoffmann et al. 2015
						Placebo	TACE + placebo	26				
48	Hsu et al. [67]	HCC	CTS	Taiwan	1	No intervention	No intervention	300	Evaluation of the influence of the mini nutritional assessment on functional status and QoL	Unclear	EORTC QLQ-C30	
49	Huang et al. [37]	HCC	NRCT	China	1	Interventional	RFA	121	Patients with a HBV associated solitary HCC with diameter of 3 cm or less underwent RFA vs. hepatic resection	Primary	FACT-Hep	
						Surgical	Hepatic resection	225				
50	Jie et al. [68]	HCC	CTS	China	1	No intervention	Informed patients	126	QoL in patients informed vs. uninformed of their diagnosis	Primary	EORTC QLQ-C30	
						No intervention	Uninformed patients	92				
51	Johnson et al. [69]	HCC	RCT	26 countries	173	Medical	Brivanib	577	Brivanib vs. placebo as first-line therapy in patients with unresectable, advanced HCC	Secondary	EORTC QLQ-C30	
						Placebo	Placebo	578				
52	Kensinger et al. [70]	HCC	NRCT	USA	1	Surgical	LT for HCC with "MELD exception points"	106	Liver transplantation for HCC ± "exception points" vs. liver transplantation without HCC	Primary	SF-36	
						Surgical	LT for HCC without "MELD exception points	33				
						Surgical	LT without HCC	363				
53	Kirchhoff et al. [71]	HCC	RCT	Germany	5	Interventional	Transient transarterial chemoocclusion	35	Transient transarterial chemoocclusion (TACO) using degradable starch microspheres (DSM) vs. transarterial chemoperfusion without DSM	Secondary	EORTC QLQ-C30	
						Interventional	Transarterial chemoperfusion	35				
54	Koeberle et al. [72]	HCC	RCT	Switzerland, Austria	8	Medical	Sorafenib + Everolimus	59	Patients with unresectable or metastatic HCC and Child–Pugh ≤ 7 liver dysfunction	Secondary	EORTC QLQ-C30 + LASA by Bernhard	
						Medical	Sorafenib	46				
55	Kolligs et al. [73]	HCC	RCT	Germany	2	Interventional	Selective internal radiation therapy (SIRT)	13	SIRT vs. TACE in unresectable HCC	Primary	FACT-Hep	
						Interventional	Transarterial chemoembolization (TACE)	15				
56	Kondo et al. [74]	HCC	CCS	Japan	1	Interventional	Percutaneous ethanol injection therapy (PEIT) or RFA	97	QoL in patients receiving PEIT or RFA vs. QoL in patients with chronic liver disease who had neither current evidence nor history of HCC	Primary	SF-36	
						No intervention	Chronic liver disease	97				
57	Kudo et al. [75]	HCC	RCT	20 countries	154	Medical	Levatinib	478	Levatinib vs. Sorafenib as first-line treatment in patients with unresectable HCC	Secondary	EORTC QLQ-C30 + EORTC QLQ-HCC18	
						Medical	Sorafenib	476				
58	Kuroda et al. [76]	HCC	NRCT	Japan	1	Medical	Branched-chain amino acid—enriched nutrition	20	BCAA-enriched nutrition vs standard diet	Secondary	SF-8	
						No intervention	Standard diet	15				
59	Lee et al. [77]	HCC	NRCT	Taiwan	1	Surgical	Hepatic resection	121	Hepatic resection vs. TACE vs. PEI vs. best supportive care	Primary	EORTC QLQ-C30 + WHOQoL-BREF	
						Interventional	TACE	31				
						Interventional	Percutaneous ethanol injection (PEI)	8				
						No intervention	Best supportive care	1				
60	Lee [164]	HCC	CTS	South Korea	1	Mixed	Mixed treatments	40	QoL in patients receiving different types of treatments	Primary	SF-12	
61	Lei et al. [78]	HCC	NRCT	China	1	Surgical	Liver transplantation	95	LT vs. hepatic resection	Primary	SF-36	
						Surgical	Hepatic resection	110				
62	Li et al. [79]	HCC	NRCT	China	1	Interventional	High intensity focussed ultrasound therapy (HIFU) + best supportive care	151	HIFU vs. best supportive care	Unclear	QOL-LC	
						No intervention	Best supportive care	30				
63	Li et al. (2013)	HCC	RCT	China	1	Medical	TACE + Celecoxib + Lanreotide	133 (total)	TACE + Celecoxib + Lanreotide vs. TACE + Celecoxib	Unclear	EORTC QLQ-C30	
						Medical	TACE + Celecoxib					
64	Li et al. [80]	HCC	CTS	China	1	No intervention	No intervention	472	Evaluation of the prognostic value of QoL	Primary	EORTC QLQ-C30 + EORTC QLQ-HCC18	
65	Liu et al. [81]	HCC	CTS	China	2	Surgical	Hepatic resection + thrombectomy	65	Hepatic resection + thrombectomy vs. chemotherapy	Unclear	FACT-Hep	
						Medical	Systemic therapy	50				
66	Llovet et al. [82]	HCC	RCT	21 countries	121	Medical	Sorafenib	303	Sorafenib vs. placebo in patients with advanced HCC who had not received previous systemic treatment	Secondary	FHSI-8	
						Placebo	Placebo	299				
67	Lv et al. [83]	HCC	RCT	China	1	Medical	Parecoxib	60	Parecoxib vs. placebo in HCC patients receiving TACE	Unclear	Questionnaire by Lv	
						Placebo	Placebo	60				
68	Manesis et al. [84]	HCC	RCT	Greece	1	Medical	Triptorelin + Tamoxifen	33	Triptorelin + Tamoxifen vs. Triptorelin + Flutamid vs. placebo	Secondary	Spitzer QoL Index	
						Medical	Triptorelin + Flutamid	23				
						Placebo	Placebo	29				
69	Meier et al. [85]	HCC	CTS	USA	1	No intervention	No intervention	130	Qol in patients with therapy naive HCC and liver cirrhosis	Unclear	EORTC QLQ-C30 + EORTC QLQ-HCC18	
70	Meyer et al. [86]	HCC	RCT	Great Britain	1	Interventional	Transarterial chemoembolization: with cisplatin	44	TACE vs. TAE	Secondary	EORTC QLQ-C30 + EORTC QLQ-HCC18	
						Interventional	Transarterial embolization	42				
71	Mihalache et al. [87]	CCA	CTS	Romania	1	Mixed	Curative + palliative treatments: surgery, stenting, chemotherapy, drainage etc	133	QoL in patients with curative and palliative treatment for CCA	Unclear	EORTC QLQ-C30	
72	Mikoshiba et al. [88]	HCC	CTS	Japan	1	Mixed	Different treatments	192	Validation of the Japanese version of EORTC QLQ-HCC18	Primary	EORTC QLQ-C30 + EORTC QLQ-HCC18 + FACT-Hep	
73	Mikoshiba et al. [89]	HCC	CSS	Japan	1	No intervention	Depressive Symptoms	36	QoL in HCC patients with or without depressive symptoms	Primary	EORTC QLQ-C30 + EORTC QLQ-HCC18	
						No intervention	Without depressive symptoms	91				
74	Mise et al. [90]	HCC	CTS	Japan	1	Surgical	Hepatic resection	108	QoL in patients receiving hepatic resection for HCC	Primary	SF-36	
75	Montella et al. [91]	HCC	CTS	Italy	1	Medical	Sorafenib	60	Sorafenib in patients > 70 years of age with advanced HCC	Unclear	FHSI-8	
76	Müller et al. [92]	HCC	RCT	Austria	1	Interventional	Octreotide + PEI	31	Octreotide + PEI vs. Octreotide	Unclear	VAS by Priestman & Baum	
						Medical	Octreotide	30				
77	Norjiri et al. [93]	HCC	RCT	Japan	1	Medical	Branched-chain amino acid (Aminoleban EN) supplementation	25	Branched-chain amino acid enriched nutrition vs. standard diet in HCC patients with up to 3 tumour nodules < 3 cm receiving RFA	Secondary	SF-8	
						No intervention	Standard diet	26				
78	Nowak et al. [94]	HCC	CTS	Australia	13	Medical	Octreotide	46	Octreotide	Primary	FACT-Hep + Pt DATA Form	Part of a larger Phase II trial (Cebon J, et al. Br J Cancer 2006; 95: 853–61.)
79	Nugent et al. [95]	HCC	RCT	USA	1	Interventional	Stereotactic body radiation therapy	12	SBRT vs. TACE as bridging therapy before liver transplantation for HCC	Secondary	SF-36	
						Interventional	TACE	15				
80	Ortner et al. [96]	CCA	RCT	Germany, Switzerland, Austria	4	Interventional	Photodynamic therapy + Stenting	20	Photodynamic therapy + Stenting vs. Stenting	Secondary	EORTC QLQ-C30	
						Interventional	Stenting	19				
						Interventional	Non-randomized PDT + Stenting	31				
81	Otegbayo et al. [97]	HCC + CCA	CTS	Nigeria	1	No intervention	Unclear	34	QoL in patients with HCC	Primary	WHOQoL-BREF	
82	Palmieri et al. [98]	HCC	CCS	Italy	1	No intervention	HCC	24	QoL in patients with HCC vs. CLD vs. healthy controls	Primary	SF-36	
						No intervention	CLD	22				
						No intervention	Healthy controls	20				
83	Park et al. [117]	CCA	RCT	South Korea	1	Interventional	Photodynamic therapy + S-1	21	Photodynamic therapy ± S-1 for patients with unresectable hilar cholangiocarcinoma	Secondary	DDQ-15	
						Interventional	Photodynamic therapy	22				
84	Poon et al. [99]	HCC	CTS	China	1	Surgical	Hepatic resection	66	Hepatic resection vs. TACE	Primary	FACT-G	
						Interventional	TACE	10				
85	Poon et al. [100]	HCC	RCT	China	1	Medical	TACE plus branched-chain amino acid as supplement	41	Branched-chain amino acid enriched nutrition vs. standard diet in HCC patients with unresectable tumour	Secondary	FACT-G	
						No intervention	Standard diet	43				
86	Qiao et al. [101]	HCC	CSS	China	1	No intervention	No intervention	140	QoL and TNM stage in patients with HCC	Primary	FACT-Hep	Drop-out 2 patients for disease progression. 3 patients excluded as > 5 items missing
87	Ryu et al. [102]	HCC	CSS	South Korea	1	No intervention	High symptom scores	53	Effect of symptoms on QoL in patients with HCC	Primary	FACT-Hep	
						No intervention	Low symptom scores	127				
88	Salem et al. [103]	HCC	NRCT	USA	1	Interventional	TACE	27	TACE vs. 90Y radioembolization	Primary	FACT-Hep	
						Interventional	Radioembolization	29				
89	Shomura et al. [104]	HCC	CTS	Japan	1	Medical	Sorafenib	54	QoL during sorafenib treatment	Primary	SF-36	
90	Shun et al. [105]	HCC	CTS	Taiwan	2	Interventional	Stereotactic radiation therapy	99	QoL during SRT treatment for HCC	Primary	FLIC	
91	Shun et al. [106]	HCC	CTS	Taiwan	1	Interventional	TACE	89	QoL during TACE for HCC	Primary	SF-12	
92	Somjaivong et al. [107]	CCA	CSS	Thailand	2	No intervention	No intervention	260	Evaluation of the influence of symptoms, social support, uncertainty and coping on QoL	Primary	FACT-Hep	
93	Steel et al. [108]	HCC	NRCT	USA	1	Interventional	Hepatic arterial infusion with 90Y-Micosphere	14	90Y-Microsphere vs. Cisplatin during hepatic arterial infusion for HCC	Primary	FACT-Hep	Butt et al. 2014 and Steel et al. 2006 report on the same data
						Interventional	Cisplatin infusion of Cisplatin into the hepatic artery	14				
94	Steel et al. [109]** (1)**	HCC	CCS	USA	1	No intervention	HCC	21	Evaluation of the influence of sexual functioning on QoL	Secondary	FACT-Hep	
						No intervention	CLD	23				
95	Steel et al. [110] (2)	HCC	CCS	USA	1	No intervention	HCC	82	QoL evaluation by patients themselves vs. caregivers	Primary	FACT-Hep	Steel et al. 2006 reports on the same data
						No intervention	Caregivers	82				
96	Steel et al. [111]	HCC	CCS	USA	1	No intervention	HCC	83	Comparison of QoL in patients with HCC vs. chronic liver disease vs. healthy controls	Primary	FACT-Hep	Butt et al. 2014 reports on the same data
						No intervention	Chronic liver disease	51				
						No intervention	Healthy controls	138				
97	Steel et al. [112]	HCC + CCA	CTS	USA	1	No intervention	No intervention	321	Evaluation of the prognostic value of QoL	Secondary	FACT-Hep	
98	Sternby Eilard et al. [113]	HCC	CTS	Sweden, Norway	4	No intervention	No intervention	205	Evaluation of the prognostic value of QoL	Primary	EORTC QLQ-C30 + EORTC QLQ-HCC18	
99	Tanabe et al. B[114]	HCC	CTS	Japan	1	Surgical	Hepatic resection	188	Hepatic resection for HCC	Unclear	Questionnaire by Tanabe	
100	Tian et al. [115]	HCC + CCA	RCT	China	1	Interventional	TACE with Bruceas- and iodized oil + oral injection of Ganji Decoction	49	TACE with Bruceas- and iodized oil + oral injection of Ganji Decoction vs. regular TACE	Unclear	QOL-LC	
						Interventional	TACE	48				
101	Toro et al. [116]	HCC	NRCT	Italy	1	Surgical	Hepatic resection	14	Hepatic resection vs. TACE vs. RFA vs. no treatment	Primary	FACT-Hep	
						Interventional	TACE	15				
						Interventional	RFA	9				
						Control	No treatment	13				
102	Treiber et al. [118]	HCC	RCT	Germany	1	Medical	Octreotide + Rofecoxib	32	Octreotide + Rofecoxib vs. Octreotide in palliative HCC	Primary	SF-36	
						Medical	Octreotide	39				
103	Ueno et al. [119]	HCC	CTS	Japan	1	Surgical	Impaired QoL	21	Evaluation of the factors influencing QoL after hepatic resection	Primary	Questionnaire by Ueno	
						Surgical	Preserved QoL	75				
104	Vilgrain et al. [121]	HCC	RCT	France	25	Interventional	SIRT	174	SIRT vs. Sorafenib in locally advanced and inoperable HCC	Secondary	EORTC QLQ-C30 + EORTC QLQ-HCC18	
						Medical	Sorafenib	206				
105	Wan et al. [120]	HCC	CTS	China	1	Mixed	Different treatments	105	Development and validation study of a new QoL tool	Primary	QOL-LC	
106	Wang et al. [122]	HCC	RCT	China	1	Interventional	TACE + RFA	43	TACE + RFA vs. TACE	Primary	FACT-G	
						Interventional	TACE	40				
107	Wang et al. [123]	HCC	CSS	China	1	No intervention	No intervention	277	Evaluation of the influence of symptoms on QoL	Primary	FACT-Hep + MDASI	
108	Wang et al. [124]	HCC	NRCT	China	1	Interventional	Immunotherapy + TACE or radiotherapy	42	TACE or radiotherapy with vs. without immunotherapy with DC-CTLs	Primary	EORTC QLQ-C30	
						Interventional	TACE or radiotherapy	26				
109	Wible et al. [125]	HCC	CTS	USA	1	Interventional	TACE	73	QoL after TACE for HCC compared with healthy normal values	Primary	SF-36	
110	Wiedmann et al. [126]	CCA	CTS	Germany	1	Interventional	Photodynamic therapy + biliary stent	23	PDT and biliary drainage in patients with hilar CCA	Secondary	Spitzer QoL Index	
111	Woradet et al. [127]	CCA	CTS	Thailand	5	Mixed	Mixed treatments	99	QoL in patients receiving standard or palliative therapy for HCC	Primary	FACT-Hep	
112	Xie et al. [128]	HCC	CTS	China	1	Surgical	Hepatic resection	58	Hepatic resection vs. TACE	Primary	SF-36	
						Interventional	TACE	44				
113	Xing et al. [129]	HCC	CTS	USA	1	Interventional	TACE with Doxorubicin loaded beads	118	QoL in HCC patients receiving TACE with Doxorubicin loaded beads vs. healthy norm values	Primary	SF-36	
114	Xing et al. [130]	HCC	CTS	USA	1	Interventional	Y90 radioembolization	30	QoL in patients with advanced infiltrative HCC and portal vein thrombosis receiving Y90 radioembolization vs. Healthy norm values	Primary	SF-36	
115	Xu et al. [131]	HCC	RCT	China	1	Interventional	TACE + Jian Pi Li Qi Decoction	50	TACE + Jian Pi Li Qi Decoction-Decoction vs. TACE ± placebo	Primary	MDASI-GI	
						Interventional	TACE + placebo	40				
						Interventional	TACE	50				
116	Yang et al. [132]	HCC	CTS	China	1	Mixed	Different treatments	114	Validation of the Chinese version for the EORTC QLQ-HCC18	Primary	EORTC QLQ-HCC18	
117	Yang et al. [133]	HCC	CTS	China	1	Interventional	TACE or TEA	17	Evaluation of survival and QoL in HCC patients receiving TACE or TEA therapy	Secondary	EORTC QLQ-C30	
118	Yau et al. [134]	HCC	CTS	China	1	Medical	PEGylated recombinant human arginase 1	20	QoL and survival analysis of HCC patients receiving treatment with PEGylated recombinant human arginase 1	Secondary	EORTC QLQ-C30 + EORTC QLQ-HCC18	
119	Ye et al. [135]	HCC + CCA	RCT	China	4	Medical	Shungbai San	67	Shunbai San dermal application vs. Placebo dermal application	Primary	EORTC QLQ-C30 + QOL-LC	
						Placebo	Placebo	66				
120	Yen et al. [136]	HCC	NRCT	USA	4	Medical	Capecitabine 1000 mg/m2 + PHY906 1000 mg	3	Phase I/II study of Capecitabine/PHY906 in HCC patients	Secondary	FACT-Hep	
						Medical	Capecitabine 750 mg/m2 + PHY906 600 mg	8				
						Medical	Capecitabine 750 mg/m2 + PHY906 800 mg	31				
121	Zhang et al. [137]	HCC	NRCT	China	1	Medical	Sorafenib	102	HCC patients with complete response after TACE or RFA who received sorafenib or not	Unclear	FACT-Hep	
						No intervention	No sorafenib	55				
122	Zheng et al. [138]	HCC	NRCT	China	1	Surgical	Surgical treatment	29	Surgical vs. conservative treatment of spinal metastasis in HCC patients	Primary	FACT-Hep	
						No intervention	Conservative treatment	33				
123	Zhu et al. [139]	HCC	RCT	17 countries	111	Medical	Everolimus	362	Everolimus vs. placebo	Secondary	EORTC QLQ-C30	
						Placebo	Placebo	184				
124	Zhu et al. [140]	HCC	RCT	27 countries	154	Medical	Ramucirumab	283	Ramucirumab vs. placebo	Secondary	FHSI-8 + EQ-5D	QoL data is reported in Chau et al. 2017
						Placebo	Placebo	282				

Abbreviations: *JPLQ-Decoction *Jian Pi Li Qi Decoction (mixture of Chinese medical herbs), *Shungbai San* traditional mixture of Chinese medicine containing 5 main plant-based ingredients, *Coriolus versicolor* mushroom of the family of Basidiomycota used in the traditional Asian medicine, Aminoleban *EN* mixture of amino acids, hydolysed collagen, dextran, rice oil, minerals and vitamins, *BCAA* branched-chain amino acids, *DC-CTLs* dendritic cell-cytotoxic T lymphocytes, *Ganji Decoction* mixture of Chinese medical herbs

### Health-related quality of life instruments

**Fig. 2:**
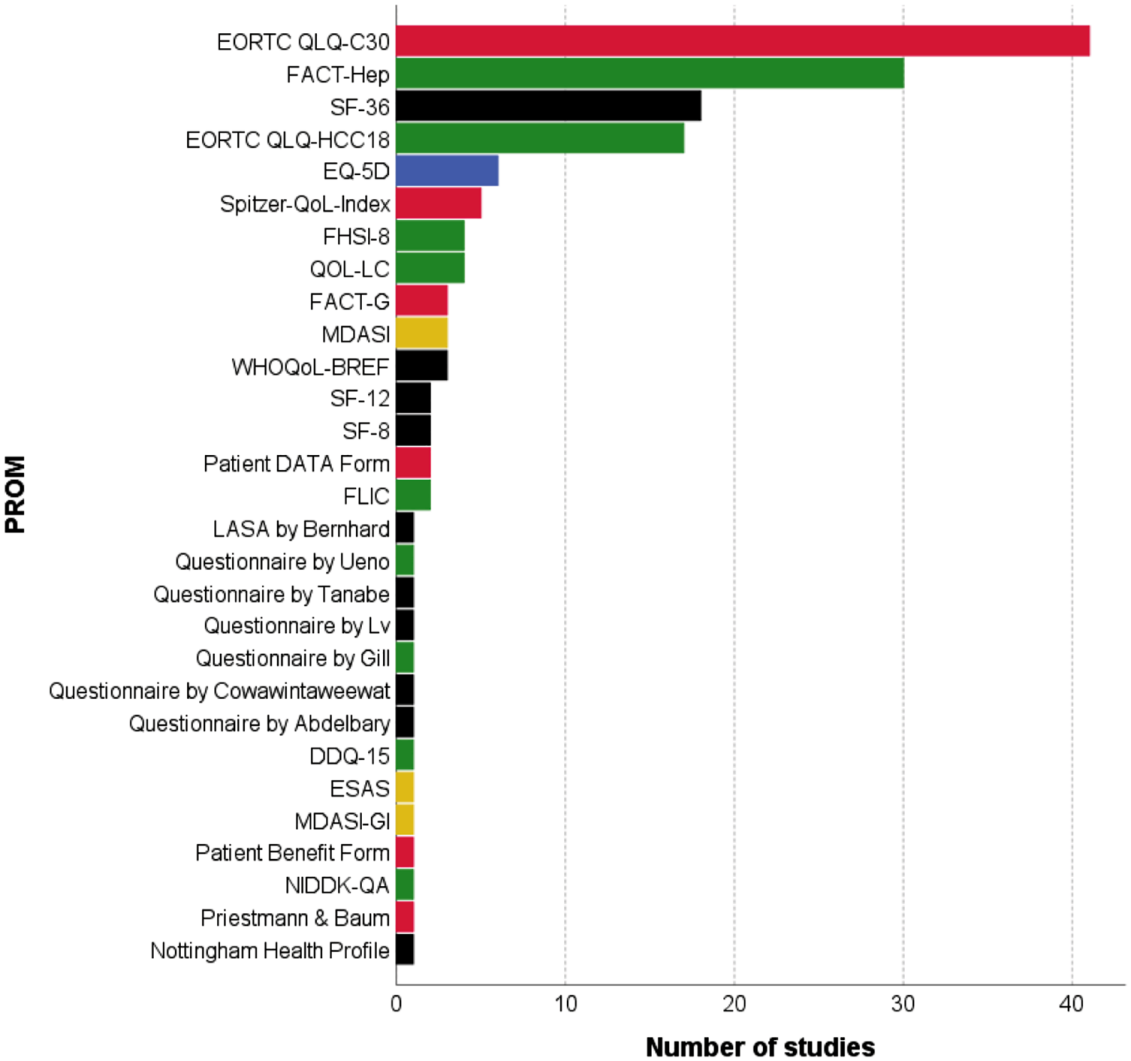
Health-related quality of life instruments used in the included studies. Generic (black), cancer-specific (red), cancer-type-specific (green), utility-based (blue) and symptom index (yellow). *EORTC* European Organization for Research and Treatment of Cancer, *EQ* EuroQol, *ESAS* Edmonton symptom assessment scale, *FACT* Functional Assessment of Cancer Therapy, *FLIC* The Functional Living Index-Cancer, *Pt DATA Form* Patient Disease and Treatment Assessment Form, *QoL* quality of life, *NIDDK-QA* National Institutes of Diabetes and Digestive and Kidney Diseases QoL Assessment, *SF* Short Form Health Survey, *VAS* visual analogue scale, *WHO* World Health Organization, *WHO-BREF* abbreviated version of the WHOQOL-100, *WHOQOL-100* WHO quality of life 100 tool

**Fig. 3:**
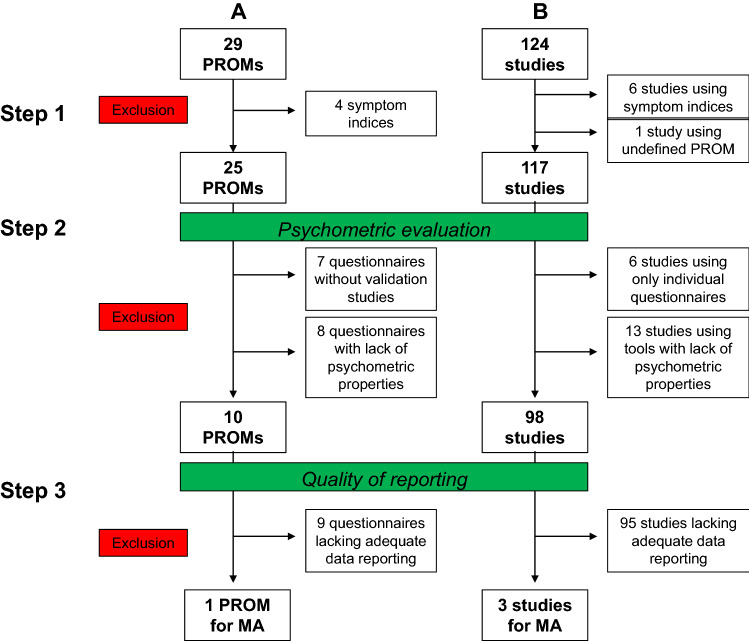
Flow chart of **a** included HRQoL measures and **b** number of studies from qualitative data analyses to quantitative data analyses. *PROM* patient-reported outcome measure, *MA* meta-analyses

In total, 29 different HRQoLs in 124 studies instruments were identified by our search (Figs. 2 and 3). Of those, 26 different HRQoL PROMs were identified in HCC patients, 8 in CCA patients and 4 different tools in mixed patient cohorts. Multiple studies used more than one HRQoL tool (Table 1). The identified instruments covered all types of HRQoL (generic, cancer-specific, cancer-type-specific and utility-based HRQoL instruments) (Fig. 2).

Despite being labelled as HRQoL instruments in the studies, a number of the identified instruments solely address cancer symptoms and, thus, lack the multidimensionality that is requested for HRQoL and were, thus, excluded from further analyses (Fig. 3 step 1). These were (a) MD Anderson symptom inventory; (b) ESAS: Edmonton symptom assessment scale; (c) MD Anderson symptom inventory – gastrointestinal and (d) FHSI-8 FACT hepatobiliary symptom index. The remaining 25 instruments (117 studies) were included in the further analyses (Fig. 3). These 25 instruments use two to eight domains covering various aspects of quality of life (e.g. physical and mental health, role functioning and symptom burden). The EORTC QLQ-C30 and the FACT-G have cancer-type-specific supplements (EORTC QLQ-HCC18 and FACT-Hep) which can only be used in combination with the more general questionnaire. The questionnaires comprise 5 (EQ-5D) to 47 questions (NIDDK-QA) and have a recall period from the 24 h (EQ-5D) to 4 weeks (SF-8/12/36, Patient Benefit Form). Most of them can be completed within 10 min.

### Methodological assessment of HRQoL instruments

The methodological quality of the remaining 25 HRQoL instruments was assessed as outlined in the methods section. Results are shown in Table 3. If no data for a given HRQoL instruments were available for HCC/CCA patients, additional Medline searches were performed to identify methodology studies that evaluated the PROM in closely related patient populations like chronic liver disease. These studies are indicated in Table 3.

**Table 3:** Overview of the methodological quality of HRQoL tools in primary liver cancer

	Psychometric properties									
References	Test–retest reliability	Internal consistency	Content validity	Criterion validity	Construct validity	Responsiveness	Acceptability	Feasibility	Floor/ceiling effects	Interpretability
Generic PROMs										
LASA** by Bernhard (Koeberle et al.)	0	0	0	0	0	0	0	0	0	+
NHP	0	+ (Bianchi 2003)	0	0	0	0	+ (Bianchi 2003)	+ (Bianchi 2003)	0	+
SF-8	0	0	0	0	0	0	0	0	0	+
SF-12	0	0	0	0	0	0	0	0	0	+
SF-36	+ (Ünal 2001*)	+ (Bayliss 1998*, Ünal 2001*, Zhou 2013*, Casanovas Taltavull 2015*)	0	0	+ (Bayliss 1998*, Ünal 2001*, Zhou 2013*)	0	+ (Bayliss 1998*, Ünal 2001*, Zhou 2013*)	+ (Ünal 2001*)	− (Bayliss 1998*, Zhou 2013*); ± (Ünal 2001*)	+
Questionnaire by Abdelbary	0	0	0	0	0	0	0	0	0	+
Questionnaire by Cowawintaweewat	0	0	0	0	0	0	0	0	0	+
Questionnaire by Lv	0	0	0	0	0	0	0	0	0	+
Questionnaire by Tanabe	0	0	0	0	0	0	0	0	0	+
WHOQoL-BREF	0	+ (Lin 2018*, Lee 2007)	0	0	± (Lin 2018*)	0	+	+	0	+
Cancer specific PROMs										
EORTC QLQ-C30	0	+ (Lee 2007)	0	0	0	0	+	+	0	+
FACT-G	+ (Yount 2002*, Zhu 2008*)	+ (Yount 2002*, Zhu 2008*)	+ (Cella 1993*)	+ (Zhu 2008*)	0	0	+	+	0	+
FLIC	0	0	0	0	0	0	0	0	0	+
Patient Benefit Form	0	0	0	0	0	0	0	0	0	+
Patient DATA Form	0	0	0	± (Nowak 2008, Cebon 2006)	+ (Nowak 2008)	− (Nowak 2008)	± (Nowak 2008, Cebon 2006)	+	− (Nowak 2008)	+
Priestman & Baum	0	0	0	0	0	0	0	0	0	±
Spitzer QoL Index	0	0	0	0	0	0	+ (Barbare 2005, Wiedmann 2004)	+ (Berr 2000, Doffoël 2008, Barbare 2005)	0	+
Cancer–type-specific PROMs										
DDQ-15	0	0	0	0	0	0	0	0	0	+
EORTC QLQ-HCC18	+ (Chie 2012, Chie 2015, Mikoshiba 2012)	± (Mikoshiba 2012, Chie 2012)	+ (Blazeby 2004*)	0	± (Mikoshiba 2012; Chie 2012, Chie 2015)	± (Chie 2012, Chie 2015)	+ (Meier 2015, Mikoshiba 2012, Fan 2013)	+ (Chie 2012, Chie 2015, Fan 2013, Meier 2015)	± (Meier 2015, Chien 2015)	+
FACT-Hep	+ (Heffernan 2002, Yount 2002*, Zhu 2008)	+ (Heffernan 2002, Steel 2006, Mikoshiba 2012)	+ (Heffernan 2002)	+ (Heffernan 2002; Zhu 2008)	+ (Heffernan 2002, Zhu 2008, Mikoshiba 2012)	+ (Steel 2006, Zhang 2015) ± (Nowak 2008)	± (Nowak 2008); + (Zhang 2015; Steel 2007; Huang 2014)	+	0	+
NIDDK-QA	+ (Kim 2000*)	+ (Kim 2000*)	± (Gross 1999*)	+ (Kim 2000*)	+ (Kim 2000*)	+ (Kim 2000*)	0	0	0	+
QOL-LC	+ (Wan 2010*)	+ (Wan 2010*)	± (Wan 2010*)	- (Wan 2010*)	+ (Wan 2010*)	+ (Wan 2010*)	+ (Wan 2010*)	+	+ (Ye 2016)	+
Questionnaire by Gill	0	0	0	0	0	0	0	0	0	+
Questionnaire by Ueno	0	0	0	0	0	0	0	0	0	+
Utility based PROMs										
EQ-5D	± (Ünal 2001*)	0	0	+ (Krabbe 2003*)	0	+ (Unal 2001*, Chau 2017)	+ (Ünal 2001*, Chow 2014, Chau 2017)	+	± (Ünal 2001*)	+

^*^Publications were identified via additional search in Pubmed. These studies were not solely conducted HCC/CCA patient populations but contain closely related patient populations like patients with chronic liver disease or extrahepatic bile duct tumours

0 = not reported (no evaluation completed),—= evidence not in favour, ±  = weak evidence, +  = evidence in favour

Gross 1999 + Kim 2000: development of questionnaire and validation of psychometric properties in patients with cholestatic liver disease/liver transplantation

^**^linear analogue-self assessment

Marked with * are studies that investigate psychometric properties in closely related patient cohorts (not only containing HCC/CCA patients). Rating: 0 no data reported;—evidence not in favour; + evidence in favour; ± conflicting evidence (rating scale adapted from [4, 5])

The most frequently evaluated dimension in all HRQoL tools was reliability (test–retest reliability and internal consistency). With a test–retest correlation of more than 0.70, adequate performance for 6 out of 12 PROMs (SF-36, FACT-G, EORTC QLQ-HCC18, FACT-Hep, NIDDK-QA and QOL-LC) was confirmed [41, 88, 120, 141–146]. For the EQ-5D, correlation coefficients ranging from 0.58 to 0.98 were observed showing that not all scales in this PROM are reliable enough [141]. Internal consistency was evaluated with the calculation of Cronbach’s α. A value greater 0.70 was considered sufficient according to COSMIN guidelines [16]. This could be observed in 8 out of 12 HRQoL tools (NHP, SF-36, WHO-BREF, EORTC QLQ-C30, FACT-G, FACT-Hep, NIDDK-QA and QOL-LC) [27, 77, 88, 120, 141, 142, 144–151]. Concerning validity, rarely all three pre-defined categories (content, criterion and construct validity) were evaluated. More frequently only one or two aspects of validity were examined. Content validity was evaluated investigating the process of questionnaire creation. In case of the FACT-G, FACT-Hep and EORTC QLQ-HCC18, the process described included qualitative studies with inclusion of expert opinions, patient reports and current literature [28, 144, 152]. Merely three PROMs (FACT-Hep, FACT-Hep and NIDDK-QA) were compared to the gold standard (i.e. an already established questionnaire), thus, testing criterion validity [144–146]. In order to evaluate construct validity, group comparisons using performance status (such as the Karnofsky Performance Status) were used for the EORTC QLQ-HCC18 and FACT-Hep questionnaires as it is known that a higher performance status correlates with better HRQoL [41, 88]. Construct validity within the SF-36 was evaluated using the correlation with hypothesized scores (conceptually related and unrelated scores) [141, 148, 149]. Kim et al. compared item scores between ambulatory patients and liver transplant recipients as well as examined correlations between the domain scores of NIDDK-QA vs. SF-36 and Mayo risk score, respectively [146]. The Wilcoxon signed-rank test was used by Chie et al. to evaluate if the changes in score were significant before and after treatment. For example, patients undergoing surgical treatment suffered significantly more pain compared to before which reflects an adequate responsiveness of the EORTC QLQ-HCC18 [41]. Steel et al. evaluated the clinically meaningful changes of the FACT-Hep over time and found significant decrements in all subscales from baseline to 3-month follow-up [147]. The SF-36 performed poorly during the evaluation of floor and ceiling effects with patients scoring the highest or lowest possible score in distinctly more than 15% which was the set cut-off [148, 149]. Valid acceptability and feasibility were assumed when the response rate was > 80%, or the time to complete the questionnaire was 10 or less minutes [24, 27, 46, 56, 85, 88, 120, 126, 141, 148, 149, 153]. The interpretability of all PROMs was considered acceptable as higher scores in QoL scales represent higher HRQoL, and higher scores within the symptom scales represent lower HRQoL.

Due to a lack of data concerning the basic psychometric evaluation or negative results, only the following 10 HRQoL instruments were considered methodologically adequate according to the pre-specified criteria (see methods section) and were subsequently included in further analyses (Table 3): (a) Generic HRQoL: NHP, SF-36, WHO-BREF; (b) Cancer (Condition)-specific HRQoL: EORTC QLQ-C30 and FACT-G; (c) Cancer type-specific HRQoL: EORTC QLQ-HCC18, FACT-Hep, NIDDK-QA and QOL-LC; (d) Utility (preference)-based HRQoL: EQ-5D. Only publications using one of the above-mentioned 10 HRQoL measures were included in further analyses (*n* = 98 studies) (Fig. 3 step 2).

### Quality of reporting of HRQoL data

The remaining studies were evaluated for the quality of reporting of HRQoL data. Results are summarized in Supplement 3. Of the 98 included studies, 4 (4,1%) did not specify in their methods section at what exact time points HRQoL data were measured [28, 31, 74, 79]. Many studies showed a marked discrepancy between reported HRQoL data in the results section and the frequency of HRQoL data assessment specified in the methods section. Eight studies reported only baseline HRQoL data although these trials specified in their methods section to have assessed HRQoL also during follow-up [38, 41, 42, 58, 80, 94, 98, 139]. The other 18 studies lacked reporting of HRQoL data altogether in their results section, although assessment had been announced in the methods section (supplement 3) [25, 28, 31, 44, 50, 53, 56, 66, 71, 74, 75, 80, 95, 97, 112, 134, 136, 139]. A total of 32 studies did not report raw HRQoL data and consequently could not be used for meta-analysis [21, 25, 27, 29, 32, 34, 35, 38, 40, 44–46, 49–51, 53, 56, 58, 66, 71, 75, 86, 95, 97, 112, 118, 128–130, 134, 136, 139]. The other 17 papers reported HRQoL data only in graphical form, which impedes meta-analysis [61, 64, 70, 72–74, 87, 90, 110, 113, 118, 121, 124, 128–130, 137]. Furthermore, although most studies reported the statistical methods, they used to analyse HRQoL, only 6 publications used a pre-specified statistical analysis plan addressing common methodological problems in HRQoL analysis [41, 43, 103, 104, 108, 125]. Finally, nine publications combined patient groups undergoing different treatment options (surgery/medical therapy/interventional treatment) for the reporting of HRQoL outcomes. In these cases, assignment of HRQoL outcomes to a specific treatment (surgery vs. medical therapy vs interventional treatment) was impossible [28, 42, 56, 58, 87, 88, 120, 127, 132]. In summary, only three studies remained for quantitative analyses (Fig. 3 step 3).

Supplement 4 illustrates the discrepancy between supposedly available and reported data for the FACT-Hep (A/B) and EORTC QLQ-C30 (C/D) HRQoL instruments.

### Data synthesis for HRQoL tools

For generic HRQoL instruments like the SF-36, EQ-5D or WHO-BREF, no meta-analysis following treatment was possible, either because primary data were insufficiently reported (supplement 4) or only single articles reporting raw data were identified. Similarly, for cancer (type)-specific HRQoL tools like EORTC QLQ-C30, EORTC QLQ-HCC18 and QLQ-LC meta-analysis of HRQoL data, the following treatment was impeded by either insufficient reporting during follow-up (supplement 3), or studies compared interventions that were too heterogeneous for meta-analysis. Only for the FACT-G and FACT-Hep questionnaires, clinically comparable interventions were analysed in several studies: Six studies contained surgical study groups [35, 37, 43, 81, 99, 116], two studies contained data on RFA [37, 116], and 5 studies reported extractable data in TACE patients [73, 99, 103, 116, 123]. Although FACT-G or FACT-Hep was used in several studies investigating medical treatment options for HCC, these were either single-arm studies [32, 34, 94], contained placebo control groups [31, 36, 38, 53, 137] or compared two medical treatment options [72, 136], thus, precluding a comparison to interventional/surgical treatments. Similarly, some studies used the FACT-G or FACT-Hep questionnaire to compare different interventional treatments [73, 103, 116, 122], again impeding meta-analysis. Consequently, only 3 studies using the FACT-G/FACT-Hep remained for meta-analysis (Fig. 3 step 3).

### Meta-analyses

For the comparison of surgical resection vs. TACE, only two studies reported raw data at baseline and during follow-up [99, 116] (supplement 5A). Poon et al. split the surgical cohort into two distinct subgroups: those with a complete follow-up of two years and those with a shorter follow-up. This is likely to introduce major bias as patients completing 2-year follow-up are likely to be healthier and have less aggressive tumour diseases. We, therefore, pooled the data for the two surgical groups. Supplement 5A shows the results of this exploratory meta-analysis of the mean difference in FACT-subscores (functional, physical, social and emotional well-being) at 12-month post-intervention/surgery. One additional analysis was possible: the comparison of surgery vs. RFA as data are reported in the two studies by Huang et al. and Toro et al. [37, 116]. Supplement 5B shows the results of the exploratory meta-analysis for the 12-month post-interventional/postoperative follow-up, again comparing mean differences in FACT-subscores.

## Discussion

HRQoLs represent an important domain of clinical outcomes in oncology. While definitions, implementation, evaluation and analyses of survival and toxicity/complication endpoints have been well standardized over the last decades, PROs are still under-evaluated and reported in most clinical settings. Multiple studies have aimed to define suitable HRQoL tools for different clinical settings, e.g. [4, 5], including cancer patients [6–8]. However, no concise evaluation has been performed for patients with primary liver cancers (HCC or CCA).

Although 124 studies were included in this systematic review, we were able to complete only the first two objectives of our study, namely to identify and evaluated HRQoL measures in HCC/CCA patients. However, meta-analysis of study results comparing the outcome of surgical, interventional or medical treatments for HCC/CCA patients in regard to HRQoL was barely possible due to the use of different HRQoL instruments, lack of data or insufficient reporting.

We identified 29 different HRQoL instruments, which indicate vast heterogeneity and lack of consensus in this field. Similar results have been reported before in other diseases [6–8]. Furthermore, many of the identified tools lacked basic HRQoL characteristics like multidimensionality [154, 155]. Hence many authors seemed to be unaware of the difference between mere symptom measures and HRQoL instruments. In addition, validation of HRQoL is poor for most instruments in HCC/CCA patients (Table 2). As expected, the best psychometric data were available for cancer-type-specific HRQoL instruments, like EORTC QLQ-HCC18 or the FACT-Hep. Interestingly, even for common generic and disease-specific HRQoL tools, like the Spitzer quality of life index and the EORTC QLQ-C30, data in HCC/CCA patients are sparse. Hence, evaluation of these common tools in this patient cohort seems necessary in future studies. In addition, even for HRQoL measures developed especially for liver cancer patients, psychometric properties were less stringent as might have been thought. The EORTC QLQ-HCC18 shows mixed psychometric results [41, 88]. FACT-Hep, on the other hand, although showing good psychometric properties, has been validated only in mixed patient populations including patients with liver metastases and pancreatic cancer in addition to HCC/CCA patients [144, 147]. Similarly, the preference-based HRQoL EQ-5D has been extensively evaluated in chronic liver disease, but little psychometric data are available in HCC/CCA patients. Future studies should address these shortcomings.

Nevertheless, our analysis revealed suitable HRQoL instruments with sound psychometric properties that should be used in all future HRQoL studies. These are SF-36 [156] for *generic* HRQoL measurement. The SF-36 is a generic HRQoL instrument consisting of 36 items divided into eight scales (Physical Functioning, Emotional Role Functioning, Physical Role Functioning Bodily Pain, General Health, Vitality, Social Functioning, Mental Health, Health Transition) [156]. The number of response choices per item ranges from two to six. The scores for each scale range from 0 to 100. A higher score indicates a better QOL. The time frame of the SF-36 is ‘last week’ [141].

For *cancer-specific HRQoL* measurement in HCC/CCA patients, the EORTC QLQ-C30 [157] and the FACT-G can be recommended. Both have limited, but acceptable psychometric properties in HCC/CCA patients and have been used extensively in this patient cohort. The 30-item QLQ-C30 measures five functional scales (physical, role, emotional, cognitive and social functioning), global health status, financial difficulties and eight symptom scales (fatigue, nausea and vomiting, pain, dyspnoea, insomnia, appetite loss, constipation and diarrhoea). The scores vary from 0 (worst) to 100 (best) for the global health status and functional scales, and from 0 (best) to 100 (worst) for symptomatic scales [157]. The FACT-G consists of 27 items for the assessment of four domains of QOL: (1) Physical Well-Being and (2) Socio-Family Well-Being contain seven items each; (3) Emotional Well-Being contains six items and (4) Functional Well-Being contains seven items. The time frame of the FACT-G is ‘last week’. Each item is scored on a 5-point ordinal scale, where 0 indicates not at all and 4, very much [152].

*Cancer-type-specific HRQoL* should be measured via the EORTC QLQ-HCC18 or FACT-Hep. The EORTC QLQ-HCC18 is an 18-item HCC-specific supplemental module developed to augment QLQ-C30 and to enhance the sensitivity and specificity of HCC-related QOL issues. It contains six multi-item scales addressing fatigue, body image, jaundice, nutrition, pain and fever, as well as two single items addressing sexual life and abdominal swelling. The scales and items are linearly transformed to a 0 to 100 score, where 100 represents the worst status [28, 88]. The FACT-Hep is a 45-item self-reported instrument that consists of the 27-item FACT-G (see above), and the 18-item hepatobiliary cancer subscale, which assesses specific symptoms of hepatobiliary cancer and side effects of treatment. The FACT-G and hepatobiliary cancer subscale scores are summed to obtain the FACT-Hep total score [37, 144]. The QoL-LC questionnaire shows good psychometric properties but has been developed and tested exclusively in Chinese patients, thus, limiting its generalizability. Similarly, NIDDK-QA as a cancer-type–specific *HRQoL* tool has been used in only one study and, thus, cannot be recommended currently.

For *utility-based HRQoL* measurement, the EQ-5D [158] has been identified as the instrument of choice. It fulfils basic psychometric requirements, and a sound database is available in HCC/CCA patients. The EQ-5D consists of five items (mobility, self-care, usual activities, pain/discomfort and anxiety/depression). Each item has three response categories: no problems, some problems and extreme problems. The sixth item is a global health evaluation scale, ranging from 0 (the worst imaginable health state) to 100 (the best imaginable health state). The time frame of the EQ-5D instrument is the present moment.

The quality reporting of the HRQoL results was insufficient overall. Few trials reported common methodological problems of HRQoL data like multiple testing, missing data or a priori hypothesis. Raw data were rarely reported and summarize measures (mean, median etc.) as well as follow-up regimes varied widely between studies. In addition, the methodological quality of the studies was generally poor. Thus, despite a total of 124 studies available, evidence regarding HRQoL in HCC/CCA patients is limited.

It is astonishing that reporting of HRQoL data does not seem to have improved over the last decades despite the publication of multiple guidelines and recommendations concerning HRQoL reporting. Few of the included studies fulfiled basic reporting standards for HRQoL like the ones proposed by Basch et al. [159], Staquet et al. [160], the International Society for QoL research (ISOQOL) [161] or the CONSORT—Patient-reported outcome extension [162].

These shortcomings in the methodological quality and reporting were the main reasons for the insufficient meta-analyses in our study. Studies had to be excluded at various points along the way (Fig. 3). The planned comparison of treatment options (surgery vs. medical treatment vs. interventional treatment) with regard to HRQoL can, therefore, be regarded exploratory at best. Future, high-quality HRQoL trials, adhering to basic reporting standards, are urgently needed to address these shortcomings.

One of the main strengths of the current study is the use of a comprehensive search strategy to identify all relevant publications. Furthermore, to our knowledge, this is the first study that assesses the methodological quality of HRQoL tools in HCC/CCA patients according to internationally accepted standards time [3, 15, 16] thereby identifying suitable HRQoL instruments for the use in future studies. In addition, this study can be used as an easy reference standard to identify available studies and raw data for the design and sample size calculation in future HCC/CCA trials. The transparent analysis process in this study can be regarded as a further strength.

The main limitation of our analysis is the heterogeneity of included studies, patients and trial designs. The variations in the application, analyses and reporting of HRQoL between studies made data synthesis difficult. The meta-analyses should regarded exploratory at best.

In summary, clear recommendations for generic, cancer-specific, cancer-type-specific and preference-based HRQoL instruments in HCC/CCA patients can be given. Meta-analysis of data comparing different treatment options in HCC/CC patients was severely limited due to methodological weaknesses of the included studies and shortcomings in reporting. Future trials should address these aspects and adhere to HRQoL reporting standards.

## Supplementary Information

Below is the link to the electronic supplementary material.

Supplementary file1 (DOCX 14 kb)

Supplementary file2 (EPS 27693 kb)

Supplementary file3 (XLSX 27 kb)

Supplementary file4 (PPTX 13710 kb)

Supplementary file5 (EPS 27693 kb)

## Data Availability

Not applicable.
