# Supplementary material for: Health-related quality of life in primary hepatic cancer: a systematic review assessing the methodological properties of instruments and a meta-analysis comparing treatment strategies
Source: Qual Life Res. 2021 Jul 20;30(9):2429–66. doi: 10.1007/s11136-021-02810-8 (PMC8405513; doi:10.1007/s11136-021-02810-8)
Supplement: Supplementary file 1 — Supplementary file1 (DOCX 14 kb) [file 11136_2021_2810_MOESM1_ESM.docx]

| **A** | **Patient**  **(HCC/iCC)** | 1 | carcinoma, hepatocellular.sh. |
| --- | --- | --- | --- |
|  |  | 2 | cholangiocarcinoma.sh. |
|  |  | 3 | bile duct neoplasms.sh. |
|  |  | 4 | (cancer* or carcinoma* or adenocarcinom* or malignan* or tumor* or tumour* or neoplasm*).tw. |
|  |  | 5 | (hepatocellular* or hepatic* or liver* or hepatoma*).tw. |
|  |  | 6 | 4 adj5 5.tw. |
|  |  | 7 | (ampulla of vater or bile duct or Klatskin).tw. |
|  |  | 8 | 4 adj5 7.tw |
|  |  | 9 | 1 or 2 or 3 or 6 or 8 |
|  | | | |
| **B** | **Outcome** | 10 | (HRQL or HRQoL or QL or QoL).ti,ab. |
|  |  | 11 | (Quality of life).mp. |
|  |  | 12 | (health index* OR health indices OR health profile).ti,ab. |
|  |  | 13 | (Health status).mp |
|  |  | 14 | ((patient OR self OR self*) adj5 (appraisal* or appraised or report or reported or reporting or rated or rating* or based or assessed or assessment*)).ti,ab. |
|  |  | 15 | (disability or function or functional or functions or subjective or utility or utilities or wellbeing or well being) adj2 (index or indices or instrument or instruments or measure or measures or questionnaire* or profile or profiles or scale or scales or score or scores or status or survey or surveys) |
|  |  | 16 | Patient Outcome Assessment.sh. |
|  |  | 17 | Quality of Life.sh. |
|  |  | 18 | 10 or 11 or 12 or 13 or 14 or 15 or 16 or 17 |
|  |  |  |  |
|  |  | 19 | 9 and 18 |
|  |  | 20 | (animals not humans).sh. |
|  |  | 21 | 19 not 20 |
