# Supplementary material for: Health-related quality of life in primary hepatic cancer: a systematic review assessing the methodological properties of instruments and a meta-analysis comparing treatment strategies
Source: Qual Life Res. 2021 Jul 20;30(9):2429–66. doi: 10.1007/s11136-021-02810-8 (PMC8405513; doi:10.1007/s11136-021-02810-8)
Supplement: Supplementary file 4 — Supplementary file4 (PPTX 13710 kb) [file 11136_2021_2810_MOESM4_ESM.pptx]

## Slide 1
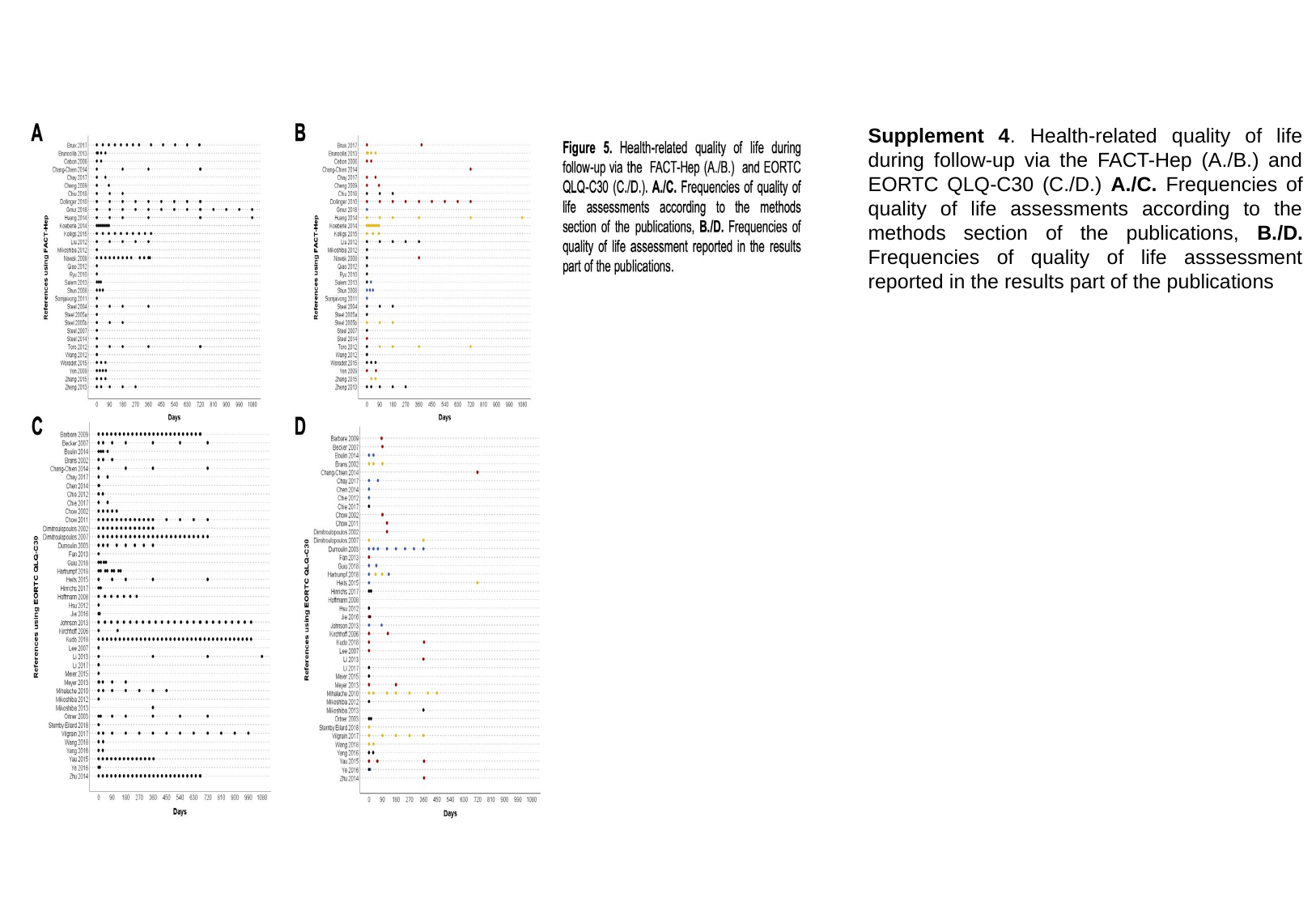

Supplement 4. Health-related quality of life during follow-up via the FACT-Hep (A./B.) and EORTC QLQ-C30 (C./D.) A./C. Frequencies of quality of life assessments according to the methods section of the publications, B./D. Frequencies of quality of life asssessment reported in the results part of the publications
